# High-Resolution Nerve Ultrasound in Adults with NF1: An Accessible and Reproducible Imaging Tool for Plexiform Neurofibromas

**DOI:** 10.3390/diagnostics15243146

**Published:** 2025-12-10

**Authors:** D. Christine Noordhoek, Koen C. van Tulder, Tessa A. Ennik, Walter Taal, Judith Drenthen

**Affiliations:** 1 Department of Neurology, Erasmus MC Cancer Institute, Dr. Molewaterplein 40, 3015 GD Rotterdam, The Netherlands; d.noordhoek@erasmusmc.nl (D.C.N.);; 2 Department of Neurology, Maasziekenhuis Pantein, Dokter Kopstraat 1, 5835 DV Beugen, The Netherlands; 3 Department of Clinical Neurophysiology, Erasmus MC, Dr. Molewaterplein 40, 3015 GD Rotterdam, The Netherlands

**Keywords:** neurofibromatosis type 1, NF1, plexiform neurofibromas, high-resolution nerve ultrasound, nerve conduction studies

## Abstract

**Background/Objectives** High-resolution nerve ultrasound (HRUS) is a promising imaging modality in patients with neurofibromatosis type 1 (NF1). The aim of this study was to evaluate the use of HRUS in adults with NF1 by assessing changes in HRUS findings over a two-year follow-up time and reporting interobserver variability. **Methods** Sixty adult patients with NF1 were invited for a study visit including a clinical examination, nerve conduction studies (NCSs) and HRUS, at baseline and after two-years follow-up. The nerve cross-sectional area (CSA) was measured at standard anatomical sites and at additional sites in cases of nerve enlargements. In 16 patients, the CSA measurements of the median nerve on one side were performed by two observers to assess interobserver variability. **Results** Fifty-two patients participated in the follow-up visit. During follow-up, 40% of nerve enlargements increased, 46% decreased and 14% remained stable. Especially larger CSA measurements at baseline showed substantial increases and decreases at follow-up. The presence or absence of plexiform neurofibromas remained the same. Interobserver agreement of median nerve CSA measurements with HRUS was 0.982 (95% CI: 0.969–0.99). **Conclusions** HRUS can be an important additional imaging tool in patients with NF1. It is helpful to distinguish between patients with and without plexiform neurofibromas, which is relevant for estimating the risk of developing malignant peripheral nerve sheath tumors (MPNSTs). The good interobserver agreement supports the use of HRUS in clinical practice. The majority of nerve enlargements decreased spontaneously in size within two years, which limits the reliability of tumor volume as sole marker for treatment response.

## 1. Introduction

Regular imaging of plexiform neurofibromas (PNs) is often needed in patients with NF1, for instance, for preoperative evaluation or distinguishing between benign and malignant lesions. PNs can cause significant morbidity such as pain and neurological deficits [1]. Furthermore, PNs can transform into malignant peripheral nerve sheath tumors (MPNST), which are a life-threatening condition [2,3]. Early detection of MPNSTs is essential, as tumor size and the presence of metastasis are associated with poor survival [4,5]. Patients with a suspicion of (symptomatic) PN regularly undergo regional MR imaging, and screening for PN tumor load with whole body-MRI (WB-MRI) is recommended in the NF1 guideline by the European Reference Network (ERN) of Genetic Tumor Risk Syndromes (GENTURIS) [6]. High-resolution nerve ultrasound (HRUS) has been suggested as a more accessible additional imaging tool with a lower carbon footprint compared to MRI [7,8,9].

Our recent study of HRUS in patients with NF1 with and without symptoms of the peripheral nervous system (PNS) reported that nerve enlargements were present in the majority of patients and can often be asymptomatic and without abnormalities in nerve conduction studies (NCSs). It was shown that a clear distinction could be made between patients with no or very few neurofibromas and patients with continuous PNs [9]. This distinction between patients with low and high tumor load could be helpful in estimating the risk for MPNST and, therefore, deciding the frequency with which patients should be monitored [10].

Longitudinal follow-up of nerve enlargements with HRUS has been reported in a group of 16 patients with NF1 [7]. Telleman et al. showed that patients with only minor HRUS abnormalities at baseline did not show a large increase in nerve size or number of nerve enlargements at 1-year follow-up. Yet in patients with severe HRUS abnormalities, both a substantial increase and decrease in nerve size were observed, which poses the question of whether these are actual anatomic changes or are related to the interobserver variability of HRUS. A study with WB-MRI showed that the majority (63%) of internal PNs reduced in size within a ten-year follow-up of adults with NF1 [11].

The aim of this study was to further evaluate the use of HRUS in a broad spectrum of adults with NF1 by assessing changes in HRUS findings over a two-year follow-up time and reporting the interobserver variability of CSA measurements. Changes in PNS symptoms and NCS findings during follow-up were also reported.

## 2. Methods

### 2.1. Study Design

Sixty adult patients with NF1 participated in the previously published cross-sectional explorative study on HRUS [9]. The baseline study visit included clinical examination, NCSs and HRUS. For this prospective longitudinal study, we invited the same participants for a follow-up visit. The follow-up visit was planned approximately 2 years after the baseline visit and consisted of the same study procedures, including clinical examination, NCSs and HRUS. The study was conducted in accordance with the Declaration of Helsinki and approved by the local medical ethics committee of Erasmus MC. All patients provided written informed consent at enrollment, which included the baseline and follow-up study visit. Adult patients with NF1, with or without symptoms related to the peripheral nervous system (PNS), were included in the study at baseline. Exclusion criteria at enrollment were having a comorbidity associated with (poly)neuropathy (e.g., alcohol abuse or diabetes mellitus) and inability to give written informed consent or to undergo HRUS and/or NCS.

### 2.2. Clinical Examination

Standardized clinical examination was performed by the patient’s treating physician during a regular visit to the outpatient clinic or by a trained neurology resident during the study visit. A short history was taken of the patients’ symptoms regarding pain and sensory and motor function. The motor function of 9 muscle groups (deltoid, biceps, triceps, wrist flexors and extensors, interossei, abductor pollicis brevis, ankle flexors, and extensors) was graded bilaterally using the Medical Research Council scale (MRC) [12]. Sensory deficits and reflexes (biceps, triceps, patellar, achilles and plantar) were documented. PNS-related symptoms were defined as either self-reported symptoms or findings at neurological examination indicating involvement of one or more peripheral nerves. Clinical findings were compared to baseline to identify new PNS-related symptoms.

### 2.3. Nerve Conduction Studies

A one-sided NCS protocol was performed on the same side as the baseline visit and by repeating the same protocol. NCSs were performed and analyzed by a trained clinical neurophysiologist or a neurology resident supervised by a trained clinical neurophysiologist. Sensory nerve action potentials (SNAPs) and sensory nerve conduction velocity of the median (3rd digit), ulnar (5th digit), radial (1st digit) and sural (ankle) nerves were recorded. Compound muscle action potentials (CMAPs), motor nerve conduction velocity, distal motor latency and F-waves of the median, ulnar, radial, fibular and tibial nerves were also recorded. Recording sites were the abductor pollicis brevis, abductor digiti minimi, extensor indicis proprius, extensor digitorum brevis and the abductor hallucis muscles, respectively. Reference values of Buschbacher were used [13]. If the NCS protocol was extended with extra tests at baseline, these were repeated at the follow-up visit. NCS findings at follow-up were compared to baseline to identify new NCS abnormalities.

### 2.4. High-Resolution Nerve Ultrasound

A two-sided standardized HRUS was performed and analyzed by a trained clinical neurophysiologist or a neurology resident supervised by a trained clinical neurophysiologist, using the Philips EPIQ Elite or Philips IU22 ultrasound machine (Philips, Eindhoven, The Netherlands), with a 4 to 18 MHz linear array transducer. The median, ulnar, radial, fibular, tibial and sural nerves were examined, as well as the brachial plexus at the truncal level. Nerve cross-sectional area (CSA) was measured manually at standard anatomical sites (Supplementary Table S1) and at additional sites in case of isolated nerve enlargements, similar to the baseline visit. Measurements at additional sites were registered with a distance in centimeters to the nearest external anatomical landmark, to ensure measuring the same site at the follow-up visit. The trunks of the brachial plexus were measured separately when possible; however, in cases where the individual trunks could not be identified, the brachial plexus was measured as a whole at both baseline and follow-up. Reference values for CSA by Goedee et al. were used [14]. Additional measurements of the radial nerve in the forearm were compared to the reference value published by Bae et al. [15]. Extreme outliers of CSA difference between baseline and follow-up were retrospectively reviewed by observers CN and JD. HRUS images were examined to verify the correct site of CSA measurement by comparing the surrounding anatomical structures. Additionally, the completeness of the measurement was compared, as was whether it was indeed performed at the inner border of the hyperechoic epineural rim. If both positioning and measurement integrity were correct, the outlier was classified as a true outlier. The Doppler technique was used to assess vascularization of the nerve at sites of nerve enlargement.

**Figure 1:**
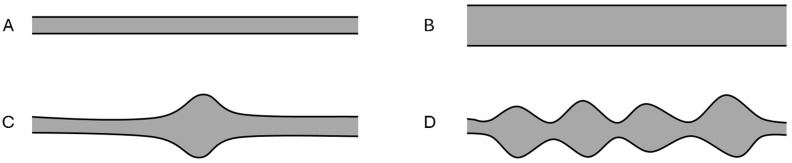
High-resolution ultrasound patterns. (**A**): Normal. (**B**): Nerve enlargement(s) with normal nerve morphology. (**C**): Focal nerve enlargement(s) with abnormal nerve morphology, such as enlarged nerve fascicles as seen in plexiform neurofibromas (focal PNs). (**D**): Continuous nerve enlargements with PN morphology (continuous PNs). Figure modified, based on Noordhoek et al. [9].

The overall pattern of HRUS findings was categorized per patient: (A) normal; (B) nerve enlargement(s) with normal nerve morphology; (C) focal nerve enlargement(s) with abnormal nerve morphology, such as enlarged nerve fascicles as seen in PN (focal PNs); (D) continuous nerve enlargements with PN morphology (continuous PNs); Figure 1 [9].

In 16 patients the CSA measurements of the median nerve on one side were performed by two observers (observer 1, CN, and observer 2, JD). The repeated measurements were taken in the same session, while the second observer was blinded for the CSA measurements of the first.

### 2.5. Statistical Analysis

Descriptive statistics were used to describe the clinical characteristics, HRUS and NCS findings. The difference in CSA between the baseline and follow-up was compared using a paired *t*-test. When comparing the difference in CSA between the baseline and follow-up across HRUS patterns, the HRUS pattern at follow-up was used. Correlation between age and CSA increase was evaluated with a linear regression model. The difference in the presence of vascularization between baseline and follow-up was compared using a chi-squared test. The intraclass correlation coefficient (ICC) for interobserver variability was calculated using a two-way random effects model for absolute agreement based on average measures. *p*-values of 0.05 were considered statistically significant, and 95% confidence intervals were reported where appropriate. Data was analyzed using R studio version 4.4.1.

## 3. Results

Between July 2021 and February 2025, 52 out of 60 patients visited our outpatient clinic for the follow-up visit. Table 1 shows the patient characteristics and results of HRUS and NCSs for the patients who participated in the follow-up visit. The median time between the baseline and follow-up visit was 26 months (range 22–40 months). Eight patients were not able to attend the second visit: six because of personal reasons, one because of a newly diagnosed brain tumor, and one patient passed away from an MPNST during follow-up.

### 3.1. Clinical Symptoms and Interventions

In total, 23 of 52 patients (44%) had PNS-related symptoms at the follow-up visit, of which 9 patients (39%) had neurological deficits at examination. Three asymptomatic patients at baseline had new PNS-related symptoms at follow-up, without corresponding HRUS changes.

Two patients had an intervention within the study period affecting one of the peripheral nerves that were examined in this study. One patient had a carpal tunnel release, and another patient had multiple corticosteroid injections in both wrists for carpal tunnel syndrome. In both cases the symptoms persisted.

One participant (age 27 years) developed an MPNST in the pelvis during the study period and passed away. At baseline, this patient had a continuous PNs pattern (high tumor load) on HRUS.

### 3.2. NCS

Thirteen patients (25%) had abnormal NCS results at the follow-up visit. Two patients with normal NCS results at baseline had new NCS abnormalities at follow-up. In both cases this could be attributed to neuropathy at compression sites. CSA measurements at those sites did not change compared to baseline. Two patients with abnormal NCS results at baseline had different NCS abnormalities at follow-up, in both cases with a corresponding increase in nerve CSA on HRUS.

### 3.3. HRUS Pattern

HRUS at follow-up was abnormal in 45 patients (87%), showing a similar distribution of patterns as at baseline: nerve enlargement(s) with normal nerve morphology in 13 patients (25%), a focal PN pattern in 9 patients (17%) and a continuous PN pattern in 23 patients (44%). All patients with a focal PN pattern and continuous PN pattern at baseline had the same HRUS pattern at follow-up. Four patients (8%) had a change in HRUS pattern between baseline and follow-up. The changes in pattern were only between normal HRUS and nerve enlargement(s) with normal nerve morphology, and the overall distribution of HRUS patterns remained the same. The change in pattern was due to fluctuations in CSA measurements that were not clinically relevant.

### 3.4. HRUS CSA Measurements

**Figure 2:**
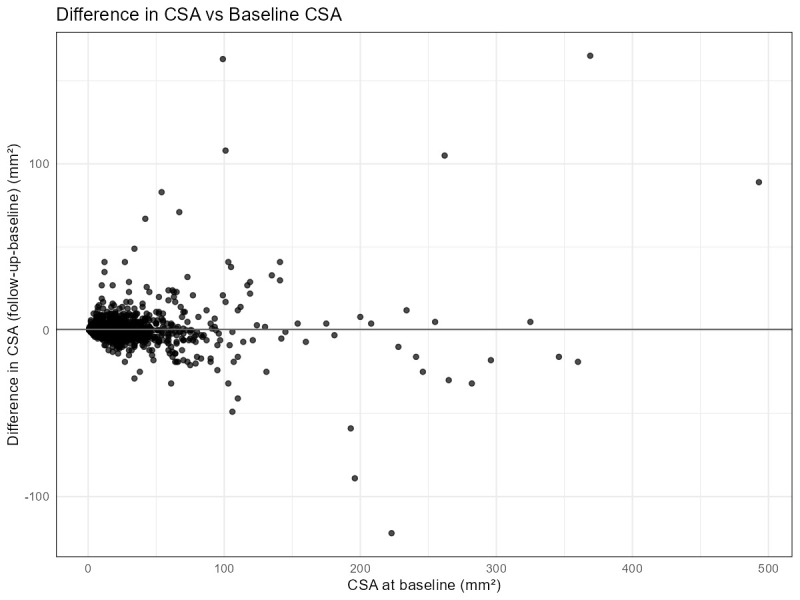
Scatterplot comparing difference in CSA between baseline and follow-up to the CSA at baseline in mm^2^. CSA = cross-sectional area.

The mean CSA difference between the baseline and follow-up of all measurements was 0.50 mm^2^ due to both increases and decreases in CSA with SD 9.33 mm^2^ (95% CI: 0.11–0.88 mm^2^, *p* = 0.012). Although this mean difference was statistically significant, a difference of 0.50 mm^2^ is not considered clinically relevant. Figure 2 shows a scatterplot comparing the difference in CSA between baseline and follow-up to the CSA at baseline. Especially larger CSA measurements at baseline show substantial increases and decreases at follow-up. A panel of individual scatterplots per nerve is included in Supplementary Figure S1.

**Figure 3:**
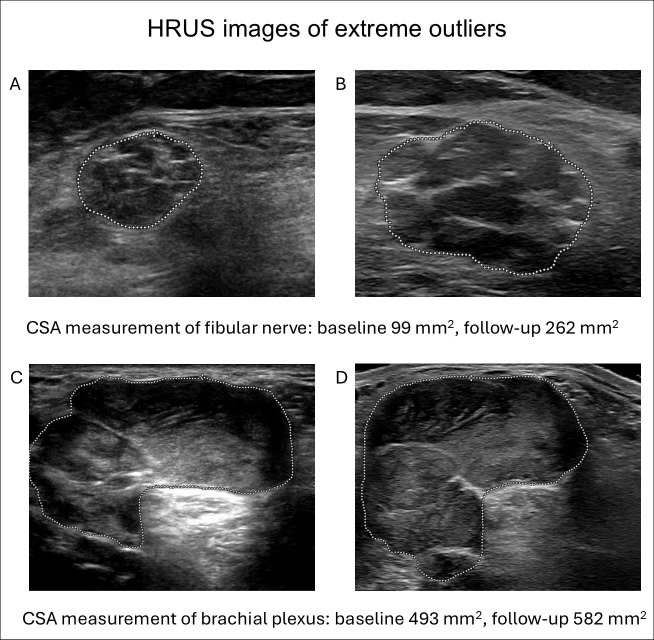
HRUS images of extreme outliers. Panels (**A**,**B**) show cross-sectional (CSA) measurements of the fibular nerve at the baseline and follow-up. The extreme difference between the baseline and follow-up was classified as a true outlier. Panels (**C**,**D**) show CSA measurement of the right brachial plexus, which was classified as a result of variation in measurement approach upon retrospective review.

Eight CSA measurements were identified as extreme outliers when compared to the baseline measurement and were subsequently reviewed. Four of these outliers reflect actual changes in nerve CSA. The remaining four outliers were attributable to a variation in measurement approach (e.g., different site of measurement or incomplete capture of the nerve CSA). Of these, three measurements involved the brachial plexus and one the median nerve in the axilla. Figure 3 shows baseline and follow-up images of two of these cases. The measurement of the fibular nerve (A and B) was classified as a true outlier, the measurement of the brachial plexus (C and D) was classified as a variation in measurement.

**Figure 4:**
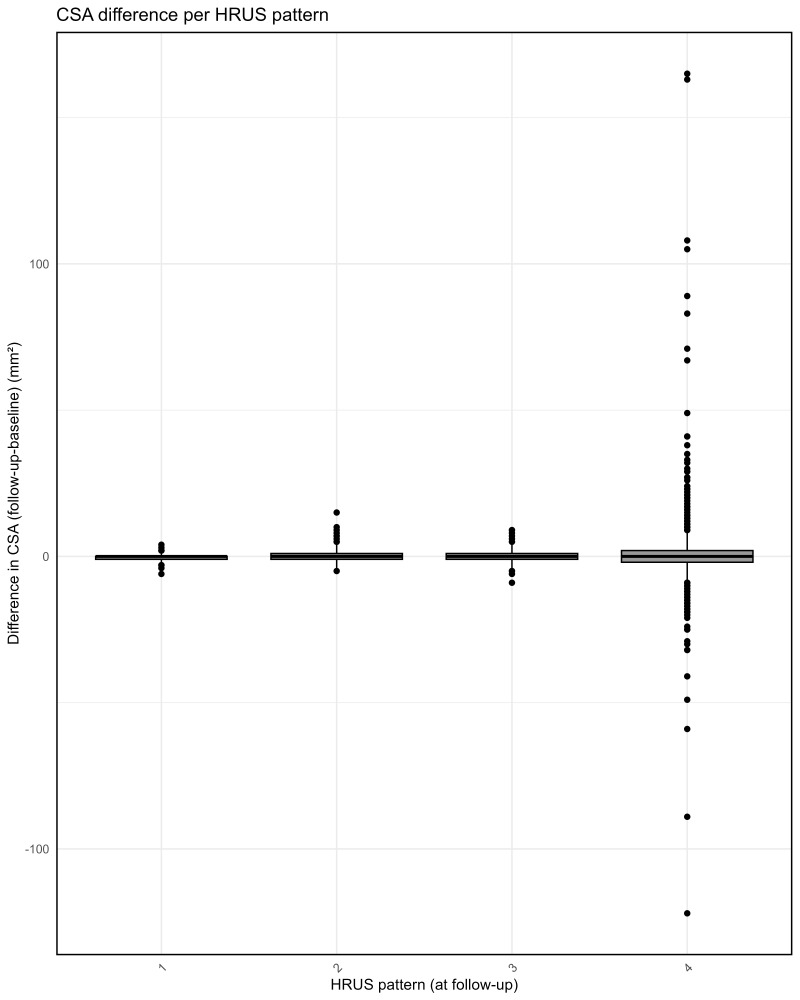
Box-and-whisker plot of difference in CSA (cross-sectional area) between baseline and follow-up per HRUS (high-resolution nerve ultrasound) pattern at follow-up. HRUS patterns: 1 normal, 2 nerve enlargement(s) with normal nerve morphology, 3 focal plexiform neurofibromas (PNs), 4 continuous PNs.

Figure 4 compares CSA differences between baseline and follow-up across HRUS patterns. Patients with a normal HRUS pattern, nerve enlargement(s) with normal nerve morphology and a focal PN pattern show only minimal differences in CSA between the baseline and follow-up. In the continuous PN pattern group, however, the mean increase is 0.76 mm^2^, with substantial variance of both increases and decreases (SD 12.63 mm^2^).

**Figure 5:**
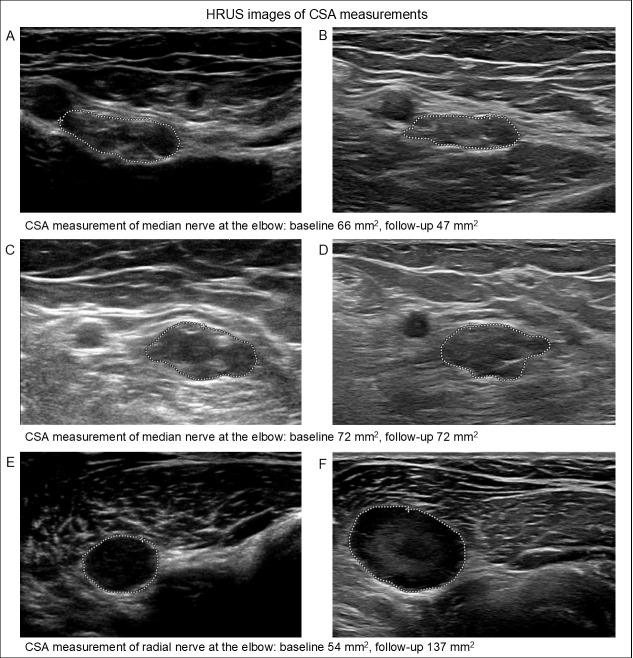
Cross-sectional area (CSA) measurements of different patients at baseline and follow-up. Panels (**A**,**B**) show a decrease in CSA, panels (**C**,**D**) a stable CSA, and panels (**E**,**F**) an increased CSA. HRUS = high-resolution nerve ultrasound.

A total of 442 paired baseline and follow-up CSA measurements were obtained at sites of nerve enlargement in 29 patients. At follow-up, 178 nerve enlargements (40%) increased. The mean increase was 8 mm^2^ (range: 1–165 mm^2^). Of the 202 nerve enlargements (46%) that decreased, the mean decrease was −6 mm^2^ (range: −0.5–−122 mm^2^). Sixty-two nerve enlargements (14%) remained stable. Most patients had both an increase and a decrease in nerve enlargements (83%). Four patients (14%) had only an increase in the nerve enlargements, and one patient (3%) had only a decrease in the nerve enlargements. None of the four patients with only an increase in nerve enlargements had new PNS symptoms at follow-up and the age range of these patients was 32 to 58 years old. The correlation coefficient of age and CSA difference between the baseline and follow-up was 0.06 mm^2^, with a *p*-value of 0.24. Figure 5 shows three examples of CSA measurements with a decrease, stable CSA and an increase.

Vascularization was present in 2.3% of all measuring sites at baseline, compared to 3.0% at follow-up (*p* = 0.22).

### 3.5. Interobserver Variability of HRUS in NF1

**Figure 6:**
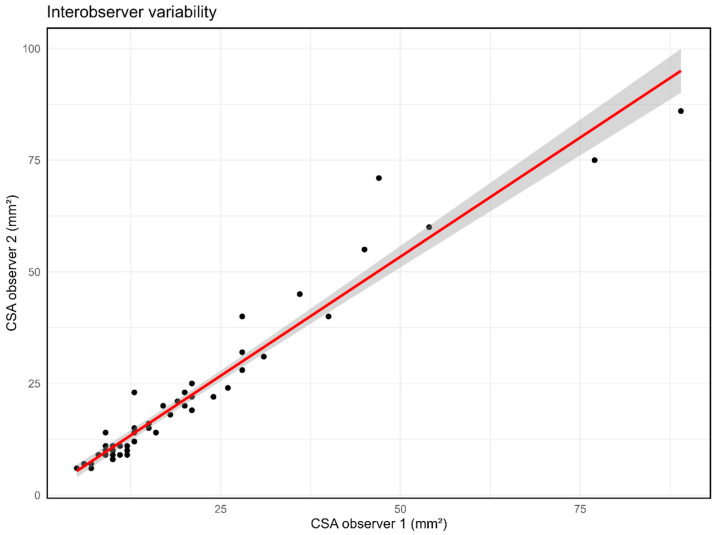
Agreement between median nerve cross-sectional area (CSA) as measured by observer 1 versus observer 2 with regression line (red) and 95% confidence interval (shading), in mm^2^.

The mean difference in CSA of the median nerve between observer 1 (CN) and observer 2 (JD) was −1.28 mm^2^ (95% CI −2.40–−0.17, SD 4.32 mm^2^), as seen in Figure 6. The ICC for interobserver variability for all CSA measurements was 0.982 (95% CI 0.969–0.99). The mean difference in CSA for only the measurements at standard anatomical sites of the median nerve was −1.38 mm^2^ (95% CI −2.76–−0.00, SD 4.62 mm^2^). The ICC for interobserver variability for CSA measurement at standard anatomical sites was 0.95 (95% CI 0.907–0.973). The mean difference in CSA for only additional measurements in case of nerve enlargements in the median nerve was −1.00 mm^2^ (95% CI −2.00–0.00, SD 3.36 mm^2^). The ICC for interobserver variability for only additional measurements was 0.995 (95% CI 0.985–0.998).

## 4. Discussion

HRUS is a promising additional imaging modality for the evaluation of peripheral nerve involvement in patients with NF1. However, the literature on HRUS in NF1 remains limited. This study provides a follow-up analysis of HRUS findings in adult patients with NF1 and reports changes in clinical PNS symptoms and NCS. To our knowledge, this is the first study to report interobserver variability for HRUS in patients with NF1.

Although the total mean CSA of all measurements increased significantly compared to baseline, the average increase of approximately 0.5 mm^2^ is not clinically relevant. More importantly, we observed that PNs can both increase and decrease in size over a two-year follow-up period, with substantial variance, especially in the continuous PN group. This pattern of bidirectional change aligns with findings from MRI studies [11,16,17] and with a small follow-up study on the use of HRUS in NF1 [7]. Interestingly, in the majority of patients with PNs, both increases and decreases in PN size were observed within the same patient. Among the four patients with only an increase in PN size, no new PNS symptoms were reported. This indicates that changes in PN size are not reliably reflected in clinical symptoms and supports the conclusion that symptom monitoring alone is insufficient to detect PN progression. Given that HRUS is a fast and relatively low-cost imaging modality, regular screening could be considered to assess whether PN growth is progressive and consistent over time, or whether apparent changes reflect natural fluctuations or measurement variability.

Although rapid PN growth is known to be associated with younger age and is typically observed in children [16], we did not find a clear correlation between age and either an increase or decrease in PN size within our adult cohort.

The percentage of patients with abnormal HRUS findings and the distribution of HRUS patterns remained unchanged over time. This suggests that, at least in the adult population, the HRUS-based phenotype is stable, and that patients with no PNs or only minor peripheral nerve involvement do not progress to develop PNs. These findings are consistent with earlier studies showing that adult patients without PNs typically do not develop them over time [7,16]. The patients with this phenotype may require less frequent follow-up to monitor for MPNST development.

Vascularization in nerve enlargements was rarely detected in our study. This did not change during follow-up time. Larger studies with longer follow-up time are needed to determine whether vascularization as detected with HRUS could be a sign of malignant transformation of PNs.

While interobserver agreement in HRUS assessments has generally been reported as good in healthy individuals and in patients with other peripheral neuropathies [18,19,20,21], these findings cannot be directly extrapolated to NF1 due to the often complex and atypical morphology of affected nerves. Accurately measuring complex, irregularly shaped structures such as PNs in a two-dimensional plane is inherently challenging and prone to considerable variability. A key strength of our study is the evaluation of interobserver agreement for CSA measurements in the median nerve of patients with NF1, which was excellent—even in the presence of PNs. Although a small but consistent difference was found between the two observers, this difference is not clinically relevant. It is, however, important to note that observer 1 was trained by observer 2, so the interobserver agreement between unrelated observers may vary.

During follow-up, eight measurements showed marked differences in CSA compared to baseline. Four of these were considered true anatomical changes, while the remaining four were attributed to variation in measurement approach. These discrepancies occurred mainly at the level of the brachial plexus, a region known for high variability in other disorders such as chronic inflammatory demyelinating polyneuropathy (CIDP) [22]. Another discrepancy was found in the axillary region. In both the axillary region and brachial plexus region, the presence of multiple nerve and vascular structures complicates HRUS measurements. The correct measurement position and angle for the brachial plexus requires skill and experience. Caution is therefore warranted when interpreting serial HRUS measurements in these areas.

Our study has several limitations. Interobserver variability was assessed only in the median nerve, while it is known that interobserver agreement with HRUS is generally poorer in the lower extremities 18. This may have led to an overestimation of the overall interobserver agreement in our study. Several limitations of the HRUS technique itself have to be acknowledged, particularly in comparison to MRI. The visualization of deeper-located lesions, such as in the trunk, is limited with HRUS due to the limited penetration depth and field of view. Additionally, with the current two-dimensional technique of measuring CSA, volumetric assessment cannot be reliably performed. Differentiating between benign and malignant lesions is also not possible with the current HRUS technique. These limitations should be considered when implementing HRUS as an additional imaging tool in clinical practice.

In conclusion, HRUS can be a valuable addition to the clinical tools we have for the evaluation of peripheral nerve involvement in patients with NF1. It is particularly useful for distinguishing between individuals with and without PN tumor load, which may aid in assessing the risk for MPNST development. The observed spontaneous decrease in PN size might limit the use of tumor volume as a sole marker of treatment response and stresses the importance of placebo control groups in therapy trials for PNs. HRUS has shown good reproducibility and interobserver agreement for measuring nerve enlargements in the median nerve. However, CSA measurements at the level of the brachial plexus and axillary region remain challenging.

HRUS, due to its limitations, cannot replace MRI, but could be suitable for use in clinical settings requiring frequent monitoring, in healthcare systems with limited MRI availability or in patients who are unable to undergo MRI without sedation. The findings of this study should be validated in prospective comparative studies involving MRI. The interobserver variability of HRUS in patients with NF1 should be further assessed in the other peripheral nerves and by independently trained observers.

## Figures and Tables

**Table 1:** Patient characteristics, *n* = 52.

	Baseline	Follow-Up
Female sex—no. (%)	40 (77)	
Median age—years (range)	44 (18–72)	
Median follow-up time—months (range)		26 (22–40)
PNS-related symptoms—no. (%)	30 (58)	23 (44)
HRUS pattern		
Normal—no. (%)	7 (13)	7 (13)
Nerve enlargement(s) with normal nerve morphology—no. (%)	13 (25)	13 (25)
Focal PNs—no. (%)	9 (17)	9 (17)
Continuous PNs—no. (%)	23 (44)	23 (44)
Abnormal NCSs—no. (%)	17 (33)	13 (25)

PNS: peripheral nervous system. HRUS: high-resolution nerve ultrasound. PN: plexiform neurofibromas. NCS: nerve conduction studies.

## Data Availability

The data presented in this study are available on request from the corresponding author due to privacy and ethical restrictions.

## Supplementary Materials

The following supporting information can be downloaded at: https://www.mdpi.com/article/10.3390/diagnostics15243146/s1, Table S1: Standard anatomical sites for measuring nerve cross-sectional area with high-resolution nerve ultrasound; Figure S1: scatterplot by nerve.
