# Supplementary material for: High-Resolution Nerve Ultrasound in Adults with NF1: An Accessible and Reproducible Imaging Tool for Plexiform Neurofibromas
Source: Diagnostics (Basel). 2025 Dec 10;15(24):3146. doi: 10.3390/diagnostics15243146 (PMC12732254; doi:10.3390/diagnostics15243146)
Supplement: Supplementary file 1 [file diagnostics-15-03146-s001.zip › Table S1 anatomical sites for measuring CSA_revised.pdf]

Supplementary Table S1: Standard anatomical sites for measuring nerve cross-sectional area with high resolution nerve ultrasound.

Nerve	Anatomical site	CSA reference values (mm²)
**Median**	Wrist	11
	⅓ Forearm	9
	½ Upper arm	10
**Ulnar**	Wrist	7
	Forearm	6
	Distal sulcus	9
	Sulcus	9
	Proximal Sulcus	9
	½ Upper arm	9
**Radial**	Upper arm	9
	Wrist (superficial nerve)	3
**Brachial plexus**	Superior trunk	8
	Middle trunk	8
	Inferior trunk	8
**Fibular**	Popliteal fossa	9
	Fibular head	11
**Tibial**	Ankle	13
**Sural**	Lower leg[a]	3

[a]10-14 cm proximal to the lateral malleolus. CSA=cross-sectional area.
