# Supplementary figures and images for: High-Resolution Nerve Ultrasound in Adults with NF1: An Accessible and Reproducible Imaging Tool for Plexiform Neurofibromas
Source: Diagnostics (Basel). 2025 Dec 10;15(24):3146. doi: 10.3390/diagnostics15243146 (PMC12732254; doi:10.3390/diagnostics15243146)

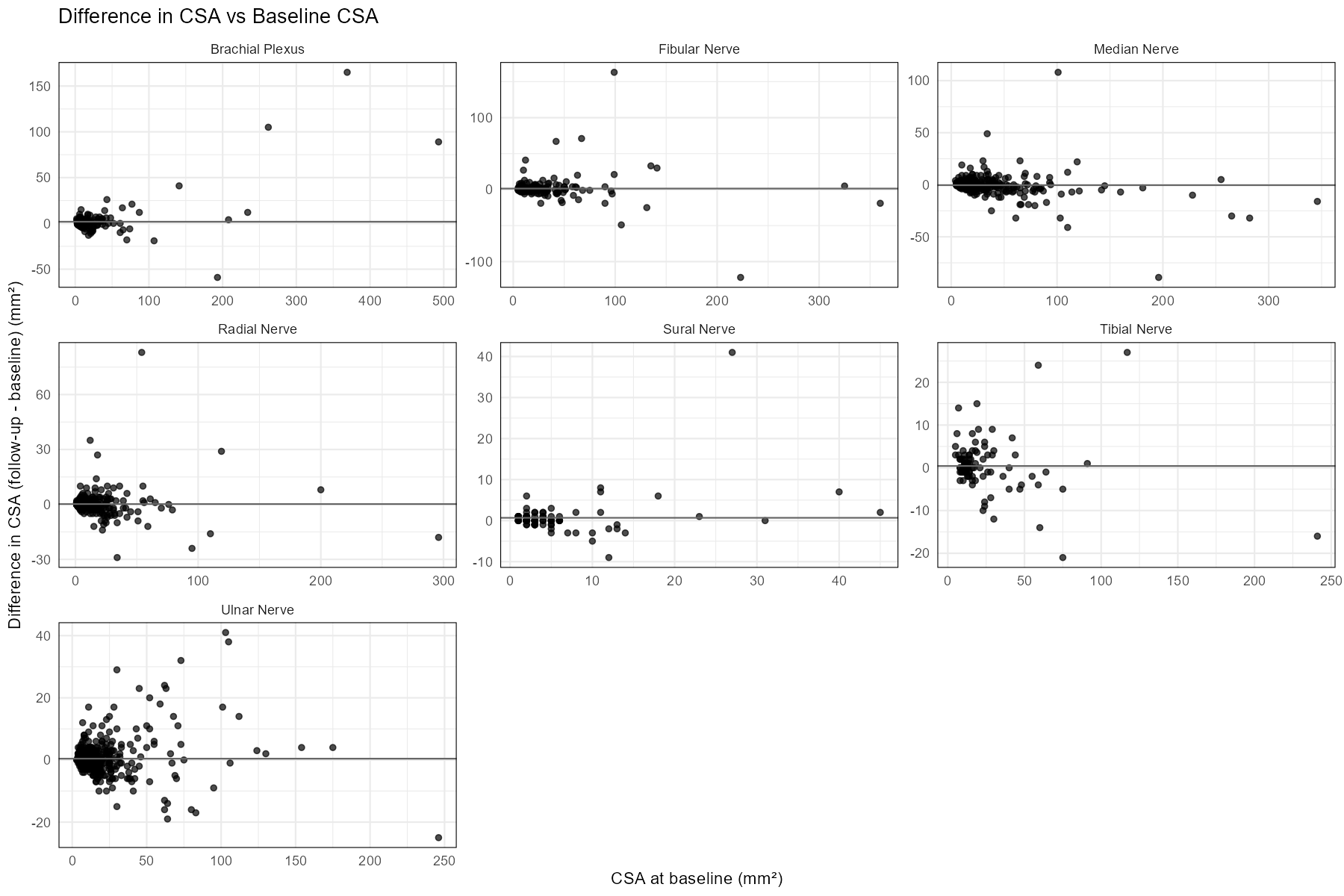

Supplement: Supplementary file 1 [file diagnostics-15-03146-s001.zip › Figure S1_scatterplot by nerve.tiff]
